# Insoluble Dietary Fibers From By-Products of Edible Fungi Industry: Basic Structure, Physicochemical Properties, and Their Effects on Energy Intake

**DOI:** 10.3389/fnut.2022.851228

**Published:** 2022-03-10

**Authors:** Baoming Tian, Yizhu Pan, Jian Wang, Ming Cai, Bangwei Ye, Kai Yang, Peilong Sun

**Affiliations:** ^1^Food Natural Product and Nutritional Health Research Center, College of Food Science and Technology, Zhejiang University of Technology, Huzhou, China; ^2^Zhejiang WisePlus Health Technology Co., Ltd, Lishui, China

**Keywords:** edible fungi, insoluble dietary fiber, energy intake, by-products, structural characterization

## Abstract

With the rapid development of the edible fungi industry in the world, especially in China, the resource utilization of edible fungi by-products has become an urgent problem for the industry's sustainable development. The waste residue of edible fungi after polysaccharide extraction by water accounts for a large proportion, which contains a large amount of water-insoluble dietary fiber (IDF). At present, the extracted residue is generally treated as fertilizer or solid waste, which not only pollutes the environment, but wastes resources too. In order to develop these by-products, expand their potential utilization in the food industry, the structure characterization, physicochemical properties, and the influence of IDF on dietary energy intake were studied. The IDF from the residues of polysaccharides extracted from four edible fungi was extracted using the Association of Official Analytical Chemists (AOAC) method. The results showed that IDF in the four kinds of edible fungi residues was similar in composition but different in texture. Cellulose and hemicellulose are the main IDF extracted from four kinds of edible fungi. Among them, *Hericium erinaceus* is the softest without obvious granular texture, following *Lentinus edodes*, while *Ganoderma lucidum* and *Grifola frondosa* have a relatively hard texture. The yield of four kinds of IDF from high to low came from *Ganoderma lucidum, Hericium erinaceus, Lentinus edodes*, and *Grifola frondosa*. Fourier transform IR (FTIR) and X-ray diffraction (XRD) spectra showed that the four IDFs had similar functional groups and all of them contained a large amount of cellulose. Physical and chemical analysis showed that all the four IDFs had certain water holding capacity, water binding capacity, and oil holding capacity. *In-vitro* digestion experiments showed that the four IDFs could inhibit the digestion of starch and fat to a certain extent. By-products of edible fungi are an ideal material for the recovery of IDFs, which have the potential to be processed into functional food materials due to their physicochemical properties and physiological functions.

## Introduction

Dietary fiber (DF) is usually not digested and absorbed by the small intestine, but serves as an important fermentation substrate and energy source for intestinal microorganisms (1). DF can be fermented by intestinal flora to produce small-molecule metabolites, which are absorbed by intestinal epithelial cells and play roles in different tissues and organs (2). The word DF refers to extremely complex carbohydrates and lignin that are difficult to digest. Eben Hipsley first described the term in his article after seeing people eating fiber-rich foods (3). The current Codex definition of DF promotes the concept that compounds that behave like fiber *in vivo*, regardless of source, can be considered DF provided that physiological health benefits can be shown (4). In general, the health benefits of DF include improved gut health, glycemic control, cholesterol reduction, weight management, and increased mineral absorption (4). DF is an edible carbohydrate polymer naturally existing in foods such as fruits, vegetables, and cereals with a degree of polymerization (DP) ≥ 3, which could neither be hydrolyzed by the digestive enzymes nor absorbed in the small intestine (1). Based on the solubility, DF can be divided into water-soluble dietary fiber (SDF) and water-insoluble dietary fiber (IDF). SDF generally includes polysaccharides, pectin, and hemicelluloses. IDF refers to the portion of DF that cannot be digested by the digestive enzymes produced in the body's digestive tract and is insoluble in hot water. IDF usually includes cellulose, insoluble hemicelluloses, and lignin.

Modern dietary habits have resulted in a serious lack of nutrients obtained from food, especially the “seventh nutrient”—DF. Although DF does not provide energy, it can effectively improve the nutritional metabolism of the human body. DFs play a significant role in improving human health and are known as potential formulations in human health due to their beneficial effects in the control of life-threatening chronic diseases, e.g., diabetes mellitus, cardiovascular disease, obesity, and cancer (3). DF is beneficial to maintain the health of intestinal flora and increase its diversity and function. The recommended daily intake of DF is 50 g, but the amount should be determined by the individual (5). Lack of DF will increase the mortality rate of colon cancer and other cancers, increase the risk of obesity/diabetes, cardiovascular diseases, inflammatory bowel diseases, and other diseases, and high intake of DF can help prevent; DF is metabolized by gut microbiota to produce short-chain fatty acids, which can regulate energy metabolism and thus prevent these diseases (5, 6). Ginseng SDF has prebiotic properties and plays beneficial roles in antioxidant status, immunity improvement, and cecal health promotion (2). Emerging evidence suggests that IDF prevents obesity by regulating gut dysbiosis, e.g., long-term insoluble yeast β-glucan supplementation ameliorated high fat diet-induced obesity, hyperlipidemia, systematic inflammation, glucose intolerance, and insulin resistance (7). IDF from defatted rice bran also played a positive role in the intervention of hyperlipidemia in high-fat diet rats (1).

The different physiological functions of DF are closely related to their structures and components. DF with strong water holding capacity, oil holding capacity, cholesterol adsorption capacity, and antioxidant properties can modify the texture of food, stabilize high-fat food and emulsions, and improve the shelf-life of foods (8). IDF plays a key role in the digestive process of the body, e.g., promoting intestinal peristalsis, increasing fecal volume, adsorption and rapid elimination of oil, heavy metals and other toxic substances, etc. (5). DF, especially IDF, may significantly affect the processing characteristics, sensory quality, and stability of various products (9). Thus, DFs are increasingly being added to foods to improve their nutritional value. The processing and manufacturing of plant-based foods, e.g., fruits, vegetables, algae, etc., is the main source of DFs. Different food processing methods also increase the DF content of food. Furthermore, its chemical and physical properties may be affected by food processing. Processing conditions also change the composition and microstructure of DFs, which, in turn, lead to desirable and undesirable effects on their physicochemical and functional properties, e.g., hydration, oil holding capacity, and viscosity (10). Therefore, DFs may also be applied as functional ingredients to improve the functional characteristics of food. Based on previous research that SDF decreased dough consistency favored cookie spread during baking and produced wider and thinner cookies; IDF (elongated and rounded) showed an opposite trend increasing dough consistency and giving rise to cookies with higher moisture, lower spread factor, and higher hardness (11). Long IDF gave rise to harder cookies with a lower spread factor. Cookies made with SDF were darker than the control cookie and cookies containing IDF. Therefore, the solubility and shape of IDF play an important role in cookie quality (11). Fiber selection will be key to enriched cookies development. Moreover, DF ingredients in processed meats might contribute to the development of healthier processed meat products (12).

In order to achieve the best activity of DF, it is necessary to consume 50–75% IDF in a daily diet, so the role of IDF cannot be ignored in inhuman health (13). At present, the application of IDF has some limitations, and strengthening the research on IDF will be of great significance to improve the application of DF in food nutrition and processing. As a large agricultural country, China should make full use of the huge domestic DF resources. If the reasonable development of the rich DF can not only make the waste into treasure, reduce the waste of resources and environmental pollution, but also has important economic and social significance. At present, the edible fungus industry develops rapidly, and edible fungus resources are abundant in the world, especially in China. Edible fungi are large fungi with high added value that can be utilized as resources. They are rich in high-quality protein, carbohydrate, various vitamins, mineral elements, and other nutrients, are characterized by high protein, low sugar, low fat, and low cholesterol, and contain a variety of bioactive substances, e.g., DF, polysaccharides, steroids, polyphenols, and most of these compounds have antioxidant, anti-tumor and other physiological functions (14). Based on previous studies about 23 kinds of edible fungi of dry weight (DW), the moisture content was 6.9–15.5 g/100 g, the ash content ranged from 1.3 to 10.1 g/100 g, the protein content ranged from 8.5 to 36.9 g/100 g, the fat content was 0.5–3.9 g/100 g, the DF content was between 14.4 and 70.2 g/100 g, the carbohydrate content is 0.5–37.3 g/100 g, the polysaccharide content was 2.1–8.3 g/100 g, and the energy is about 751–1,322 100 g/kJ (15). Mushrooms are rich in DF, yet these fibers have been overlooked by food chemists, and underutilized by the food industry, compared to other types of DF, such as fruits and vegetables (16). The DF of edible fungi was mainly composed of β-glucans, chitin, hemicellulose, and mannans. β-Glucan is a major component in edible fungus DF and it exists in both soluble and insoluble edible fiber fractions (14). The total DF percentage in DW was high in *Astraeus odoratus* (77.1%), *Schizophyllum commune* (68.2%), and *Lentinus polychrous* (60.6%). The SDF and IDF content in all the mushrooms ranged from 24.8 to 72.4% DW and 1.63 to 13.9% DW, respectively (17). The extraction of polysaccharides from edible fungi produces a large amount of insoluble residue. Normally, these residues are thrown away as waste, which not only wastes a lot of resources, but also causes pollution to the environment. Because edible fungus residue mainly comes from the fruiting body, it is rich in IDF. Therefore, extracting IDF from the residue of polysaccharides from edible fungi extracted by water plays an important role in promoting the effective and reasonable utilization of biomass resources, promoting the benign development of the edible fungi industry, and improving the economic benefits of edible fungi enterprises. At the same time, there are still few research studies on the nutritional composition and theoretical properties of edible fungus residues. In order to develop these by-products, expand their potential utilization in the food industry, IDF was extracted and characterized from four different IDF of *Lentinus edodes* (IDF-Len), *Hericium erinaceus* (IDF-Her), *Grifola frondosa* (IDF-Gri), and *Ganoderma lucidum* (IDF-Gans) by combining enzyme extraction method and chemical extraction method. The structure, hydration properties, and adsorption capacity of IDFs from these four sources were studied and the effects of starch and fat digestion models *in vitro* were studied. The indexes of IDF measured in this experiment provide a theoretical basis for future research on the properties of IDF and its influence on human energy intake, which is conducive to the development and utilization of IDF in edible fungus waste residue, and achieve the purpose of turning waste into treasure.

## Materials and Methods

### Material

The fresh *Lentinus edodes, Grifola frondosa*, and *Hericium erinaceus* were purchased from the Gouzhuang Farmers' Market in Hangzhou and pulverized into powder. *Ganoderma lucidum* extract residue was provided by Zhejiang WisePlus Health Technology Corporation Ltd. NaOH, HCl, Na_2_HPO_4_·12H_2_O, NaHPO_4_·H_2_O, sodium taurocholate, sodium carboxymethyl cellulose, DNS, and soluble starch were purchased from Sinopharm Chemical Reagent Corporation Ltd. (Shanghai, China). Neutral protease, alpha amylase, and lipase were purchased from Beijing Gao Ruisen Technology Corporation Ltd. Corn oil was purchased from Shanghai Maclean Reagent Corporation Ltd. All the other chemicals used in this study were of analytical grade.

### Preparation of Insoluble Dietary Fiber

Extraction of the IDF was carried out following the Association of Official Analytical Chemists (AOAC) 985.29 method. Fresh raw materials were washed and fully dried in a constant temperature air blowing drying oven. The dried raw materials were crushed through an 80-mesh sieve and then stored in sealed bags. Then, 1 g of crushed edible fungus powder was added to the beaker, and then 10 ml 0.25 mol/l NaOH solution was added for hydrolysis under alkaline conditions at 55°C for 2 h in a constant temperature culture oscillator, centrifuged in a centrifugal tube, removed the upper solution, and retained the lower solid for subsequent processing. The first step was to adjust the pH to between 6.3 and 6.5 with phosphoric acid buffer solution and 0.35 mol/l HCl. Then, 0.1 ml 0.4% α-amylase hydrolyzed solution was added to remove impurities, e.g., starch. The required conditions were vibration extraction at 120 rpm at 65°C for 40 min. After the supernate was removed by centrifugation, the lower residue was put into a beaker with 0.1 ml of 0.4% neutral protease solution and shaken at 120 rpm at 60°C for 90 min to remove the protein. The supernatant was removed by centrifugation again at 4,000 rpm for 15 min. Rinsed with distilled water for three times and centrifuged at 4,000 rpm to remove the supernatant from the upper layer and obtain the residue from the lower layer. In total, 280 ml of 95% ethanol was added to the prepared residue and precipitated at room temperature for 60 min to remove other impurities. The supernatant was removed by centrifugation at 4,000 rpm for 15 min, followed by decolorization with 78% ethanol for three times, and eluting with 95% ethanol until the eluent became colorless. Next, eluted IDF were pre-cooled in a ultra-low temperature refrigerator for 24 h, dried for 48 h in a vacuum freeze-dryer, crushed, weighed, calculated the yield, and stored in a refrigerator at 4°C (18).

The yield was calculated after preparation, and the formula was as follows:


$$\begin{array}{l} \text{Yield }\left(\text{\%}\right)=\frac{\text{IDF quality after extraction and drying}}{\text{Quality of raw materials before extraction}} \end{array}$$

### Scanning Electron Microscopy (SEM)

The microstructure and morphology of four IDFs were observed by a SEM (SU8010, Hitachi High Technologies Corporation, Tokyo, Japan). A small amount of dried and constant weight IDF samples of *Lentinus edodes, Hericium erinaceus, Grifola frondosa*, and *Ganoderma lucidum* were laid on the sample table for adhesion and gold plating and then the gold-plated samples were put into the SEM for scanning. The microscopic morphology of IDF was observed under an accelerated voltage of 5 kV and representative photos were taken to obtain SEM images of 500 and 1,000X.

### Fourier Transform IR Spectroscopy

The FTIR spectroscopy spectra of IDF samples of *Lentinus edodes, Hericium erinaceus, Grifola frondosa*, and *Ganoderma lucidum* were measured by a Nicolet iS5 Fourier transform infrared spectrometer (Thermo Fisher Scientific, Massachusetts, USA). First, 100 mg KBr was mixed with four different 5 mg IDF samples, and the mixture was ground and pressed. After the compression, the sample was analyzed with a resolution of 4 cm^−1^. The scanning time of the sample was 32 s and the scanning range was 400–4,000 cm^−1^. Meanwhile, the scan results of four IDFs were compared with the blank KBr background.

### X-Ray Diffraction Analysis

The crystal structures of four IDF powders were determined by an X-ray diffractometer (UItima IV, Rigaku, Japan). The DF samples from different edible mushrooms were dried to constant weights and evaluated according to the previous method (8). X-ray diffraction (XRD) was determined as follows: test voltage 40 kV, step size 0.02, test current 40 mA, scanning rate 3/min, and scanning range 6–70°.

### Determination of Water Holding and Binding Capacities

According to the previous method (19), the holding water and binding water of the four samples (IDF-Len, IDF-Her, IDF-Gri, and IDF-Gan) were measured. First, 0.5 g of IDF sample (as sample weight, W_S_) was mixed with 25 ml distilled water in a centrifuge tube at room temperature for 2 h. After centrifugation at 3,000 rpm for 10 min, the excess was absorbed with filter paper and weighed (wet weight, W_2_). The wet sample was then dried in an oven at 110°C to constant weight and weighed (DW, W_1_).

The calculation formula of holding power is as follows:


$$\begin{array}{l} \text{Water holding capacity }\left(\text{g}/\text{g}\right)=\frac{{\text{W}}_{2}-{\text{W}}_{\text{s}}}{{\text{W}}_{\text{s}}} \end{array}$$

The calculation formula of binding power is as follows:


$$\begin{array}{l} \text{Water binding capacity }\left(\text{g}/\text{g}\right)=\frac{{\text{W}}_{1}-{\text{W}}_{2}}{{\text{W}}_{2}} \end{array}$$

### Determination of Oil Holding Capacity

The oil holding capacity of four IDFs was measured by the method of Japan Gan with appropriate modifications (20). First of all, 1 g of IDF powder sample (W_S_ as the sample weight) was accurately measured into the centrifugal tube, and 5 g of corn oil was added into it, shaken at 37°C for 1 h, and centrifuged at 8,000 rpm for 30 min. Next, the supernatant was removed and the residue was collected for weighing (W_0_).

The calculation formula of oil holding capacity is calculated according to the formula below:


$$\begin{array}{l} \text{Oil binding capacity }\left(\text{g}/\text{g}\right)=\frac{{\text{W}}_{0}-{\text{W}}_{\text{s}}}{{\text{W}}_{\text{s}}} \end{array}$$

### Fat Digestion Experiment *in vitro*

*In-vitro* fat digestion experiment was carried out according to previous methods (21, 22) with slight modifications. First, 500 mg of IDF sample was mixed with 30 ml emulsion (containing 0.5% corn oil and 0.1% emulsifier) and stirred at 37°C for 30 min. Then 3.5 ml bile extract (187.5 mg bile extract dissolved in 3.5 ml phosphate buffer, pH 7.0) and 1.5 ml salt solution was added to the emulsion mixture at 37°C, stirred and adjusted to pH 7.0. 2.5 ml of freshly prepared lipase suspension was added to the mixture. After 30, 60, and 120 min, the pH of the mixture was kept at pH 7.0 by dropping NaOH solution (0.1 mol/L), and the release of free fatty acids (FFA) was monitored by measuring the volume of NaOH solution (0.1 mol/l). The above *in-vitro* fat digestion model without IDF samples was used as a control. The following equation was used to calculate the amount of FFA (%) released: $\text{FFA}\left(\%\right)=100*\left[\frac{\left({\text{C}}^{*}{\text{V}}^{*}\text{MW}\right)}{\left(2^{*}\text{M}\right)}\right]$;C (mol/L): Molality of NaOH solution; V (L): Volume of NaOH solution consumed used to neutralize the released FFA for titration of a sample; MW (872 g/mol): Average relative molecular weight of corn oil; M (g): The initial mass of corn oil; two represents the assumption that all the triacylglycerols are hydrolyzed to two molecules of FFA and one molecule of monoacylglycerol.

### Starch Digestion Experiment *in vitro*

The starch digestion experiment *in vitro* was conducted according to the methods (22–24). First, 200 mg of IDF powder was accurately weighed and added into a 10 ml starch solution (4 g/100 ml) with 40 mg α-amylase. In total, 200 ml of distilled water was prepared and put into a dialysis bag with a molecular weight of 8,000–14,000 Da for dialysis at 37°C. After incubation for 10, 30, 60, and 120 min, the dialysate samples were taken once, and the glucose content in the dialysate was determined by 3, 5-dinitrosalicylic acid. At the same time, the enzyme-starch mixture system without adding IDF powder was used as a control.

### Statistical Analysis

All results were carried out in triplicate, expressed as mean ± SD and analyzed by one-way analysis of variance followed by Duncan's multiple range tests, and the value of *p* < 0.05 was considered as statistical significance using SPSS software. The preparation of the figures was carried out in Excel and Origin software.

## Results and Discussion

### Preparation and Yield of IDF of Four Edible Fungi

**Figure 1:**
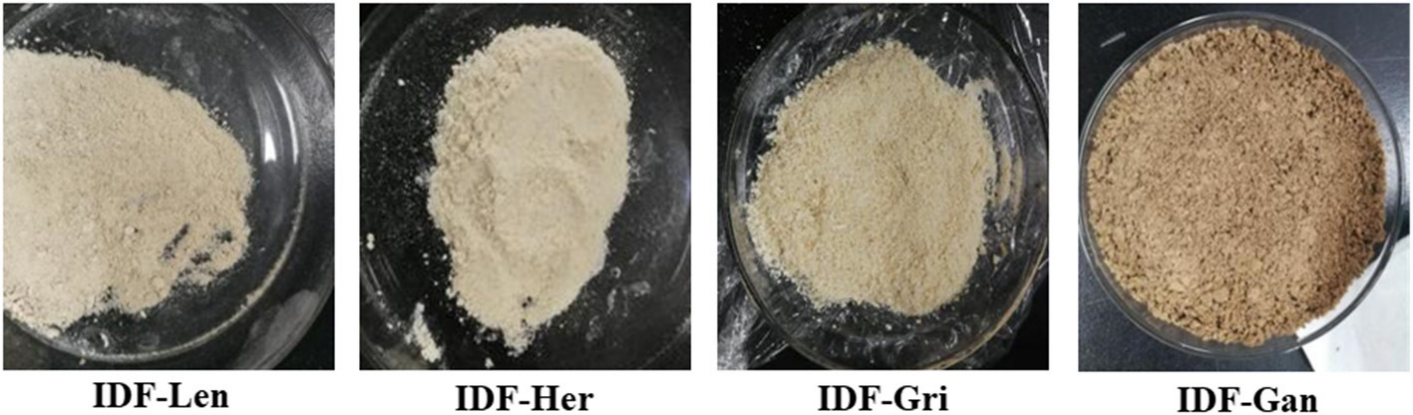
Insoluble dietary fiber (IDF) (from left to right: IDF-Len, IDF-Her, IDF-Gri, IDF-Gan).

The four IDF images extracted by AOAC985.29 recommended method are shown in Figure 1. The fiber powder of IDF-Len was light brown in color, soft in the hand, and no particle sensation; the solid powder of IDF-Her was yellow and white, and felt the softest among the four kinds of IDF powder of edible fungi; IDF-Gri powder was yellow, had a slight sense of granular, slightly brittle texture; the last IDF-Gan was the highest extraction rate, showing obvious brown, soft texture, but there were obvious lumpy.

**Figure 2:**
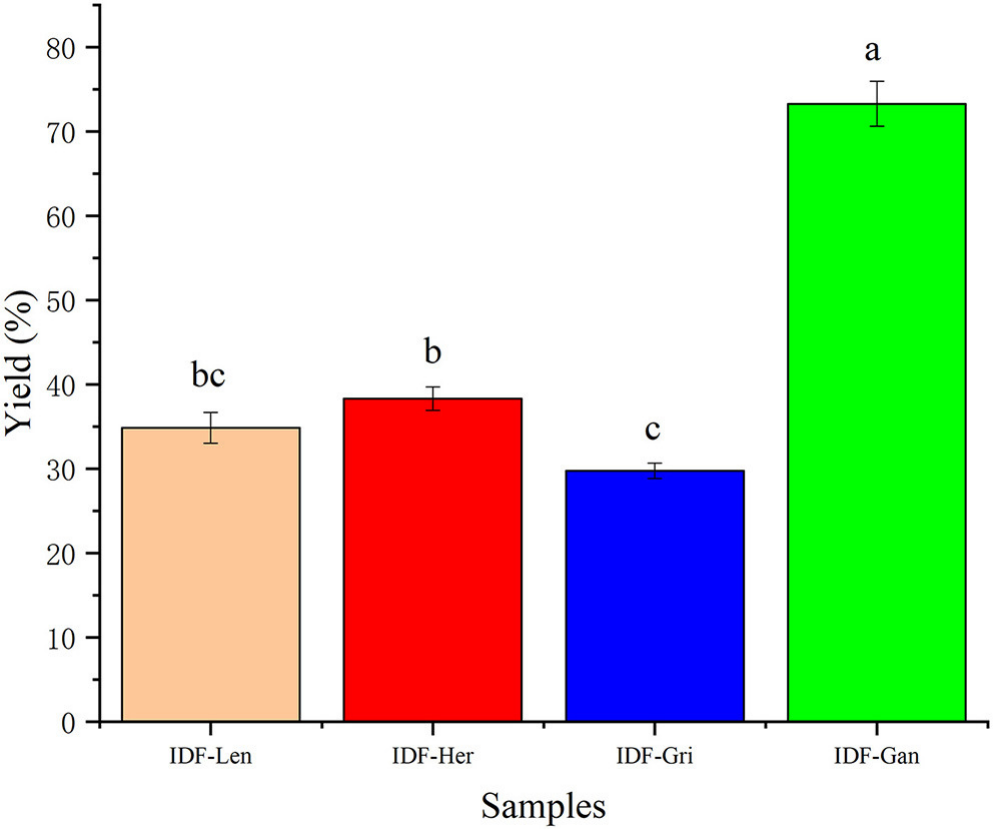
Yield of IDF of four kinds of edible fungi. Different lowercase letters indicate significant differences between the two experimental groups (*p* < 0.05).

The yield of the four IDFs is shown in Figure 2. The yield was as follows: IDF-Gan > IDF-Her > IDF-Len > IDF-Gri. The yield of IDF-Gan was significantly higher than that of the other three IDFs (*p* < 0.05), and the yield of IDF-Gri was the lowest and significantly lower than that of IDF-Gan and IDF-Her (*p* < 0.05), which may be related to the high content of IDF in *Ganoderma lucidum* and low content of IDF in *Grifola frondosa*.

### Scanning Electron Microscopy of IDF of Four Edible Fungi

**Figure 3:**
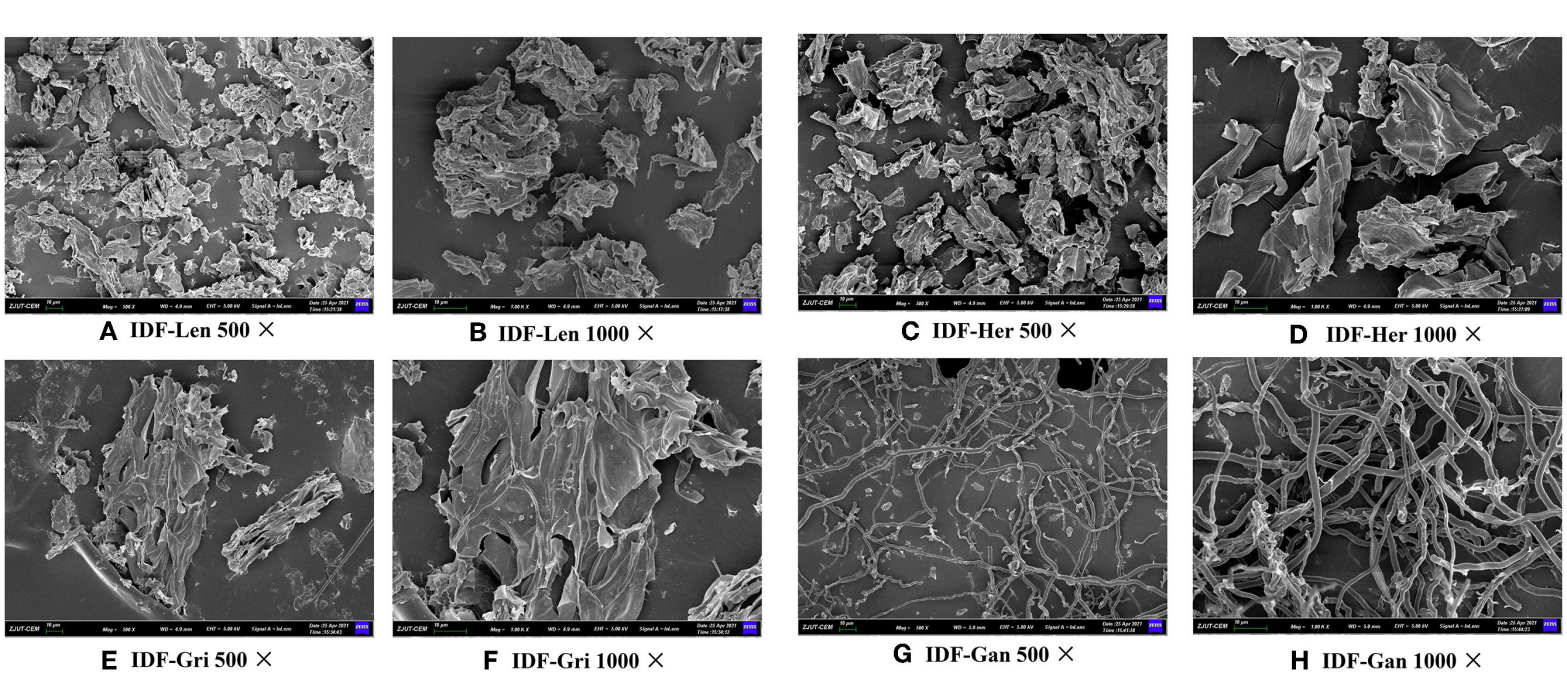
Observation of ultrastructure of IDFs from four different edible fungi by scanning electron microscopy. **(A)** IDF-Len 500X, **(B)** IDF-Len 1,000X, **(C)** IDF-Her 500X, **(D)** IDF-Her 1,000X, **(E)** IDF-Gri 500X, **(F)** IDF-Gri 1,000X, **(G)** IDF-Gan 500X, **(H)** IDF-Gan 1,000X.

As shown in Figure 3, the results of SEM showed that the IDF from the four sources have different morphologies, while the surfaces of the IDFs were loose and porous with a complicated spatial network structure, smooth and composed of and composed of small particles similar to that observed other IDFs (8, 13, 25). Under 500 and 1,000X electron microscopy, IDF-Len and IDF-Her were similar in shape, showing small pieces and uneven in size. Compared with the IDF-Her, the IDF-Len was more densely stacked. Under 500 and 1,000X electron microscopy, the morphology of IDF-Gri was long and lumpy, relatively loose. IDF-Gan showed interwoven hyphae and long filamentous morphology under 500 and 1,000X electron microscopy, with relatively stable and similar morphology, and relatively compact morphology under 1,000X diagram, similar to IDF extracted from other mushrooms (26). Previous studies on the SEM of IDF of mushrooms have been limited. The DF structure of DF extracted from *Agrocybe cylindracea* was compact or porous structures and contained porous surface, and filiform structures were mostly presented on its surface (27). Enoki mushroom IDF presents the fibrous loose structure of different sizes (25). IDF of other non-mushrooms was reported more frequently, carrot IDF surface presents irregular flakes and loose porous structure, and oat IDF is in the form of smooth and porous spatial structure (25). The loose and porous network structure increased specific surface area and was conducive to the adsorption and retention of some molecules, and could improve its water, oil, heavy metal, and salt ion absorption capacities (13, 25). Therefore, it is speculated that these four IDFs may have high adsorption capacity.

### Fourier Transform IR Spectra of IDF of Four Edible Fungi

**Figure 4:**
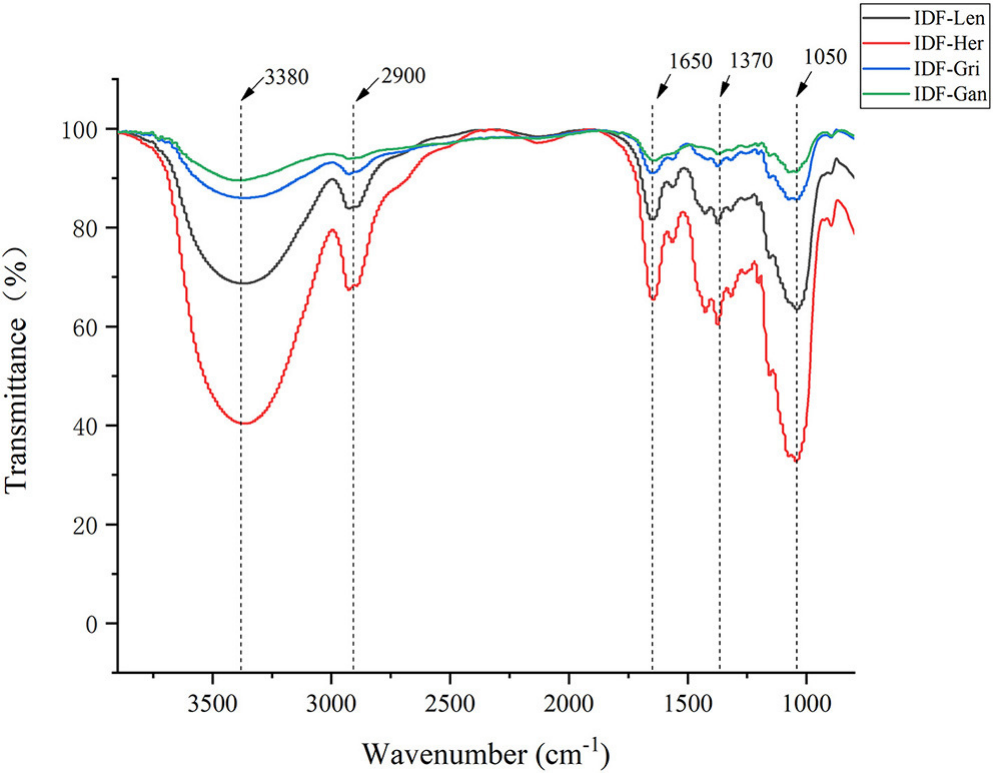
Fourier transform IR spectra of four IDFs.

Polysaccharide functional groups of IDFs were revealed by FTIR spectrum analysis (Figure 4). The four samples have obvious characteristic peaks along with the five wavelength ranges of 1,050, 1,370, 1,650, 2,900, and 3,380 cm^−1^ and these characteristic peaks mostly belong to the functional groups of insoluble cellulose. The characteristic peak at the edge of 1,050 cm^−1^ is most likely due to the C–O bond (28) in the cellulose and hemicellulose contained in IDF, while the characteristic peak near 1,370 cm^−1^ belongs to the characteristic peak of C–H bond vibration. Then, the characteristic peak near 1,650 cm^−1^ may be due to the presence of C=O double bond (29) in glucuronic acid, which can produce an absorption effect on infrared. The characteristic peaks near 2,900 cm^−1^ should be the absorption peaks of –CH and –CH_2_ from self-cellulosic polysaccharide compounds (30). The last absorption peak of 3,380 cm^−1^ is mainly from the characteristic peak caused by the vibration of –OH and hydrogen bonds in cellulose and hemicellulose (31, 32). These characteristic peaks indicated that during the extraction of IDF, the hydrophilic groups and various functional groups such as hydroxyl, carboxyl, and aldehyde groups did not change significantly. These IDFs had the typical functional groups of cellulose polysaccharides. The physical and chemical properties of these groups provided the material basis for the properties of water-holding, binding water, and oil-holding capacity of IDF.

### X-Ray Diffraction of IDF of Four Edible Fungi

**Figure 5:**
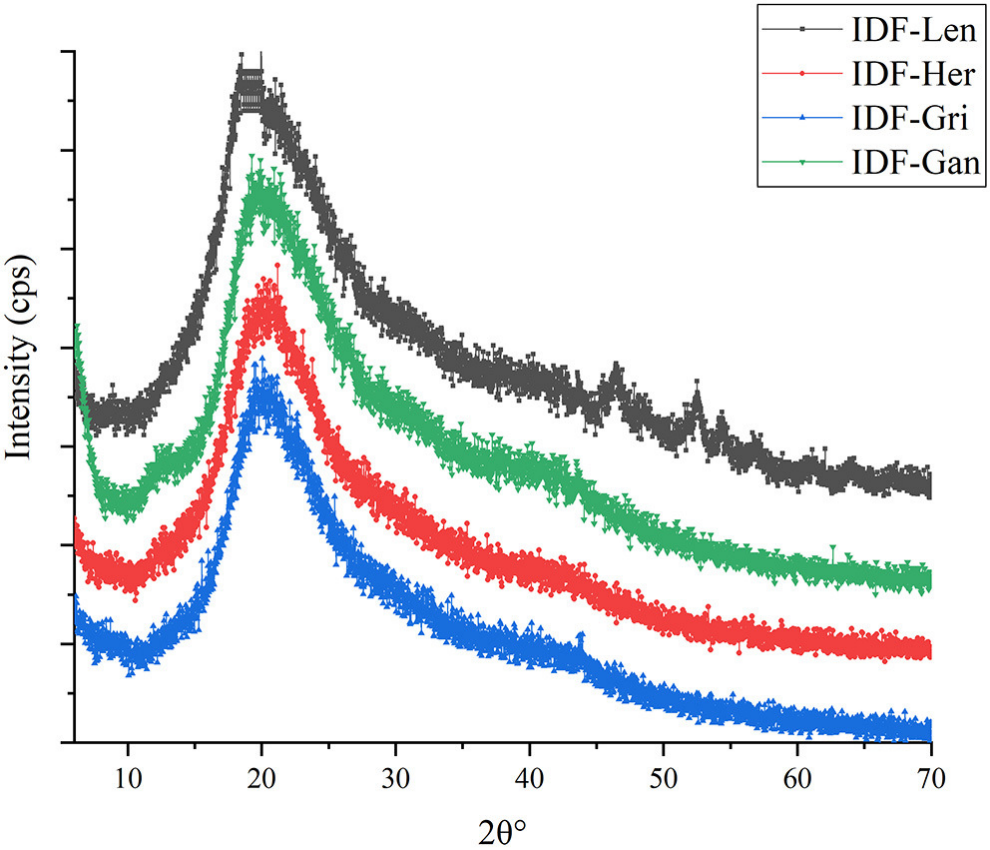
X-ray diffraction spectra of IDF of edible fungi.

An X-diffraction diagram of IDF of four edible fungi is shown in Figure 5. The four IDFs have very obvious absorption peaks between 15 and 25°, which belong to the exclusive characteristic peaks of cellulose crystal structure (33). The obvious diffraction peak of about 20° is the cellulose-type structure (34). All these conclusions indicate that the IDF extracted from the four kinds of edible fungi contains the majority of cellulose content.

### Water Holding and Binding Capacities of IDF of Four Edible Fungi

It can be concluded from Table 1 that the water holding capacity of the four IDFs is as follows: there is a consistent order between the holding and binding forces. The results of SEM showed that the structure of IDF-Gri was mostly massive, with relatively large surface area, loose and porous, which may lead to its maximum holding and binding hydraulic power among the four IDFs. These results were consistent with those of previous studies demonstrating that the IDF of bamboo shoot and citrus peels had stronger water holding capacity, oil holding capacity, and swelling capacity than SDF (8). The increased adsorption capacities of IDF can be attributed to its longer branch chains, bulkier structure, and larger gaps than the corresponding SDF (8). IDF-Len and IDF-Her have similar structures and shapes, both of which are small and dense chunks, so there is little difference in the capacity of holding water and binding water. IDF-Gan and the other three have the largest morphological differences, which are fibrous filamentous, densely intertwined, and the contact area is relatively small, so it presents a relatively low holding power and binding power. In the process of significant difference analysis of water holding capacity, there was no significant difference between IDF-Gan and IDF-Her (*p* > 0.05), i.e., their water holding capacity was similar. In the process of significant difference analysis of combined hydraulic power, the significant difference was only found between IDF-Gri and the other three IDFs (*p* < 0.05), i.e., the water holding capacity of IDF-Gri was the strongest. There were no significant differences among IDF*-*Len, IDF*-*Her, and IDF-Gan (*p* > 0.05), i.e., there was no difference in water binding power between them. The comparable water binding capacities among four SDFs might be attributed to the nature of the water-binding sites, structure, and chemical composition, which is worth further study.

**Table 1:** The water hold capacity and water binding capacity of the four insoluble dietary fibers (IDFs).

**Sample**	**Water holding capacity (g/g)**	**Water binding capacity (g/g)**
IDF-Len	3.81 ± 0.14^b^	0.13 ± 0.01^b^
IDF-Her	3.18 ± 0.17^c^	0.12 ± 0.01^b^
IDF-Gri	5.30 ± 0.15^a^	0.21 ± 0.02^a^
IDF-Gan	3.07 ± 0.11^c^	0.10 ± 0.01^b^

*The mean value in the same column with different letters was significantly different (p < 0.05)*.

### Oil Holding Capacity of IDF of Four Edible Fungi

**Figure 6:**
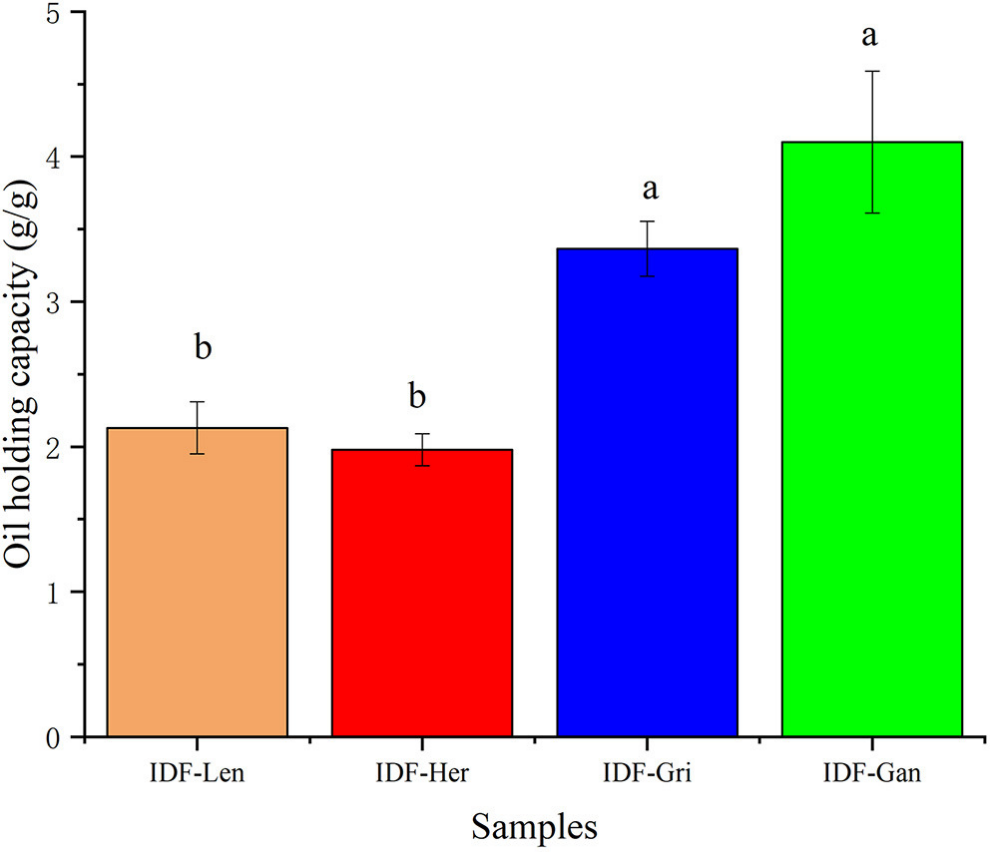
Oil holding capacity of four IDFs. Different lowercase letters indicate significant differences (*p* < 0.05) in the two experimental groups.

The chemical structure of DF contains a large number of hydrophilic groups, but fewer lipophilic groups, which is closely associated with the high water holding capacity, high oil holding capacity, and high swelling capacity of DF (8). DF with fat trapping ability can eliminate fat from the body, thus reducing fat absorption. Therefore, intake of DF is conducive to the prevention and treatment of obesity. As can be seen from Figure 6, the four IDFs showed different adsorption capacities for corn oil. IDFs have different oil adsorption capacities, among which IDF-Gan may be filamentous and dense, which is easier to adsorb oil, leading to its oil holding capacity being relatively the highest among the four. According to previous studies that the IDF had stronger water holding capacity, oil holding capacity, and swelling capacity than SDF. It may also be due to the high purity of IDF in the extract (8). While IDF-Len and IDF-Her are mostly dense clumps with relatively weak oil adsorption capacity. The IDF-Gri, though lumpy, has a relatively large surface area, so it has a higher oil holding capacity than the IDF-Len and IDF-Her. There was no significant difference (*p* > 0.05) between IDF-Gan and IDF-Gri, and between IDF-Len and IDF-Her, while the oil holding capacity of IDF-Gan and IDF-Gri was significantly higher than that of IDF-Len and IDF-Her.

Common hydrophobic groups include C–H stretching (–CH_3_ or =CH_2_), ethers (C–O), etc. A large number of hydrophobic groups were exposed during the IDF preparation *via* the enzymatic method (35), which was completely consistent with the results of the structural analysis in this study, the exposure of the above hydrophobic groups of IDFs enhances its oil holding capacity (24). This is consistent with the previous FT-IR Spectrum analysis results. The high excellent oil holding capacity is an important basis for the functional function of IDF, which can prevent fat from being overly absorbed in the intestine (24).

### Fat Digestion Experiment of Four Edible Fungi IDF *in vitro*

**Figure 7:**
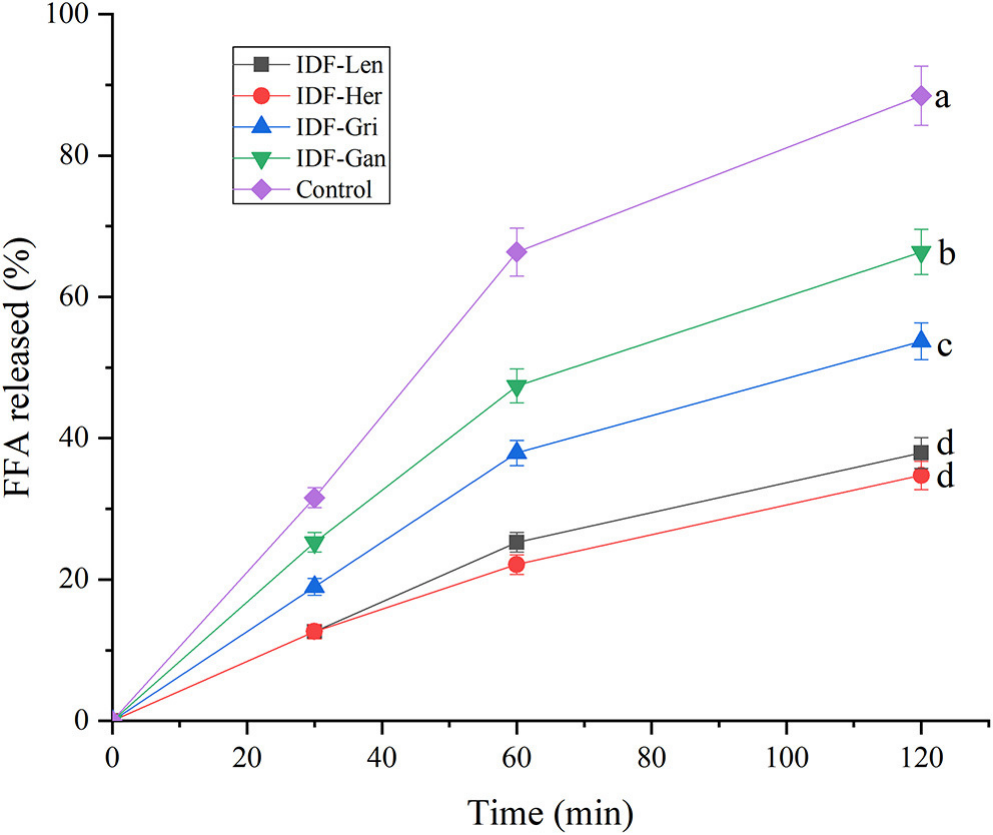
The effect of IDF on fat digestion. Curves with no letter in common are significantly different between the two treatment groups (*p* < 0.05).

As shown in Figure 7, four IDFs have different degrees of inhibition on fat digestion. In the absence of IDF, the emulsion was quickly digested completely, but in the addition of IDF, the rate of digestion decreased significantly (*p* < 0.05). Except for blank, IDF-Gan digestible emulsion was the fastest, followed by IDF-Gri and IDF-Len, and IDF-Her. The inhibitory effect of IDF-Len and IDF-Her on lipase was significantly higher than that of IDF-Gan and IDF-Gri (*p* < 0.05). In addition, the inhibitory effect of IDF-Gri was significantly higher than that of IDF-Gan (*p* < 0.05). Different DFs have different degrees of inhibition on fat digestion. It may be that the addition of IDFs blocks the effect of lipase on triacylglycerol and inhibits the effect of the enzyme, leading to the reduction of the separation of FFAs. Among them, IDF-Len and IDF-Her have the most obvious inhibitory effect, which may be because their materials are relatively soft, loose, and porous, which can combine to affect more oil and inhibit the decomposition of fat. The degree of lipid digestion was calculated by the accumulation of FFA and then titrated with NaOH, while the added IDF would limit the contact between lipase and triacylglycerol (36), thus reducing the release of FFA. This is because IDF absorbs part of the oil and cholate, blocking their contact. In addition, the specific structure, water absorption, and porous surface of IDF combine a lot of oil and cholesterol. Different IDFs have different structural characteristics and hydroscopicity, so they have different adsorption capacities for these substances, and thus have different effects on lipid digestion. The above data support that the emulsion with the addition of four IDFs had a significant inhibitory effect on lipid digestion.

### Starch Digestion *in vitro* of IDF of Four Edible Fungi

The determination results of the glucose standard curve are shown in formula: y = 0.625x-0.01070 (y represents absorption value, x represents concentration mg/ml).

**Figure 8:**
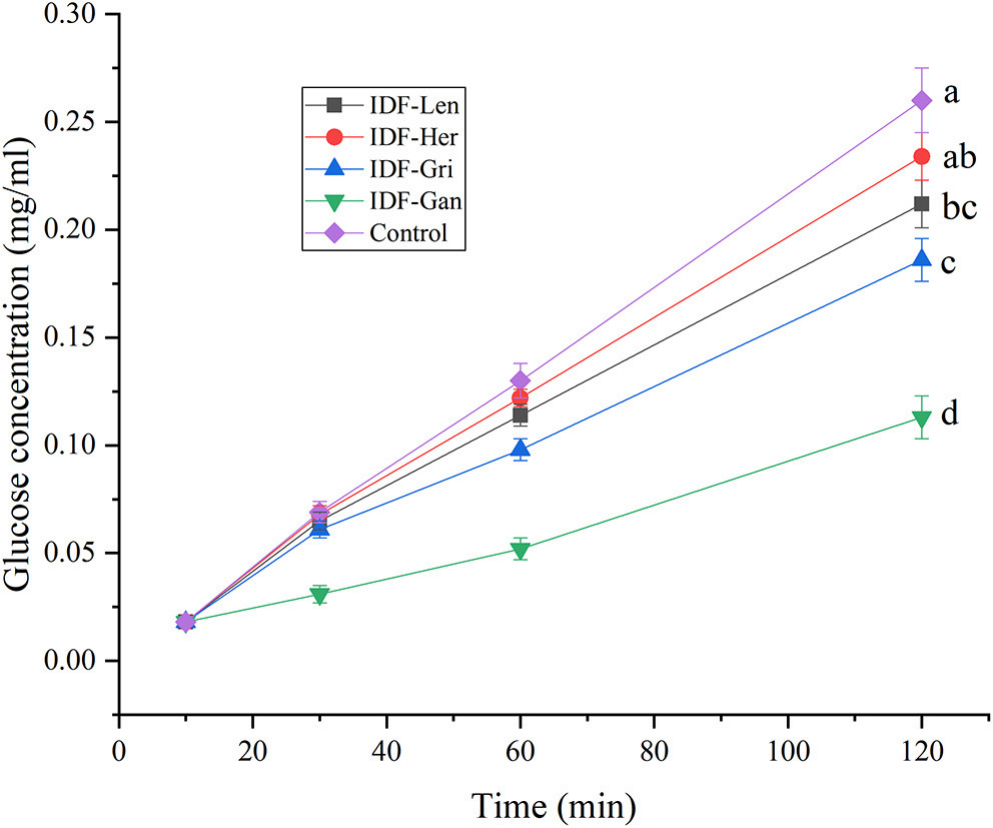
The effect of IDF on starch digestion. Curves with no letter in common are significantly different between the two treatment groups (*p* < 0.05).

According to the results of starch digestion *in vitro* shown in Figure 8, IDFs inhibit starch digestion to varying degrees. With the increase of treatment time, the digestibility of the blank group (control group without IDF) gradually increased, and the decomposed glucose content was obviously higher than that of the other four groups with IDFs. The inhibition rate of IDF-Gan, IDF-Gri, IDF-Len, and IDF-Her on starch decreased gradually and were significantly reduced by 56.54, 28.46, 18.46, and 10.00, respectively (*p* < 0.05) compared with the control group. The inhibition rate of IDF-Gan on starch was significantly higher than that of the other three IDFs (*p* < 0.05), and the starch inhibition rate of IDF-Gri was significantly higher than IDF-Her (*p* < 0.05). The mechanism of DF inhibiting α-amylase activity has been reported. According to previous reports, α-amylase non-specifically binds on the cellulose surface, and cellulose can inhibit α-amylase activity against starch (23). The possible reason is that IDF may be combined with amylase to reduce enzyme activity, or it may absorb some glucose, or IDF can inhibit the transmembrane absorption of glucose, thus inhibiting the digestion of starch, so that the decomposed glucose content is lower than that of the blank group. Moreover, IDF decreases amylase activity, which is attributed to the encapsulation of starch and enzymes by the fibers and the reduced accessibility of the enzymes to starch molecules (24). This is consistent with previous studies that IDF extracted from soybean dregs can inhibit α-amylase activity (24). Both the IDF and SDF from kiwifruit (*Actinidia deliciosa*) inhibited starch hydrolysis in a concentration-dependent manner, and IDF shows higher inhibition of α-amylase than SDF at the same concentration. Moreover, there are also studies that contradict our findings that three IDF samples (enoki mushrooms, carrots, and oats) had no effect on the digestibility of starch and would not delay the digestion of starch (25). IDF can inhibit the activity of α-amylase to some extent, which can slow down the digestive characteristics of starch food in the human body. It may be attributed to the presence of enzyme inhibitors on the surface of IDF to inhibit the activity of amylase, resulting in enzyme passivation or inactivation. The adsorption of facial-like structure on the surface of IDF reduces the substrate digestion by α-amylase. The dispersion of IDF in the system can hinder the contact between amylase and substrate, and ultimately reduce the digestibility of starch. These data indicate that the four IFDs can inhibit the activity of amylase and have the potential to be developed as a delayed digestive functional food.

## Conclusion

IDF is not able to be decomposed in the human gut and affects the moisture absorption in the digestive system. IDF plays an important role in human health and can be added to food as a functional component. At present, DF-fortified food is becoming more popular. The research on SDF has been relatively mature, while the research on IDF is relatively lack of systematic and in-depth research. The by-products of edible mushrooms are rich in a variety of carbohydrates and can be used as a valuable source of functional DF. In this study, IDFs were obtained from four different sources of edible fungi by pre-treatment with the AOAC method, analyzed the basic structure and physical and chemical properties, and preliminarily explored its effect on the digestion of starch and fat. Cellulose and hemicellulose were the main IDFs prepared from four different kinds of edible fungi. The material of *Hericium erinaceus* is the softest without obvious granular, while the material of *Ganoderma lucidum* and *Grifola frondosa* is relatively brittle. *Ganoderma lucidum* had the highest content of IDF among the four different materials. The structures of the four IDFs were different, while four IDFs share the same functional groups and contain a large amount of cellulose. The four IDFs all have good holding power and binding water power, as well as certain oil holding power. *In-vitro* fat digestion experiments and starch digestion experiments clearly showed that the four IDFs had a certain inhibitory effect on starch and fat digestion, which could be used as a bioactive ingredient in functional foods production. If four IDFs derived from edible fungus by-products can be applied to the food industry, this can not only reduce the waste of resources, turn waste into treasure, but also benefit the sustainable development of the whole industry chain of edible fungi.

Moreover, the composition and structure of IDF determine its properties. In order to expand the supplement channels of high-quality IDF, it is necessary to study the processing characteristics and health effects of IDF. In the future, IDF can be modified by physical, chemical, and enzymatic methods, improves its hydration, adsorption, ion exchange and other properties, and inhibits the activity of digestive enzymes, so as to improve its ability to regulate metabolism and protect digestive tract health. For extracting IDF, it can also be combined with chemical and enzyme extraction, which will improve the extraction rate. With the further study of IDF, there will be more new DF foods beneficial to human health.

## Data Availability Statement

The original contributions presented in the study are included in the article/Supplementary Material, further inquiries can be directed to the corresponding author.

## Author Contributions

BT: conceptualization, methodology, and writing. YP: writing—review and editing. BT and JW: review and editing. KY and BY: funding acquisition. BT and YP: investigation, software, and writing. MC: methodology. KY and PS: supervision. All authors contributed to the article and approved the submitted version.

## Funding

This study was supported financially by the Cooperative Project Fund of the Zhejiang University of Technology and Zhejiang WisePlus Health Technology Corporation Ltd. (KYY-HX-20200738). The funder was not involved in the study design, collection, analysis, interpretation of data, the writing of this article or the decision to submit it for publication.

## Conflict of Interest

BY was employed by Zhejiang WisePlus Health Technology Co., Ltd. The remaining authors declare that the research was conducted in the absence of any commercial or financial relationships that could be construed as a potential conflict of interest.

## Publisher's Note

All claims expressed in this article are solely those of the authors and do not necessarily represent those of their affiliated organizations, or those of the publisher, the editors and the reviewers. Any product that may be evaluated in this article, or claim that may be made by its manufacturer, is not guaranteed or endorsed by the publisher.
