# Supplementary figures and images for: Insoluble Dietary Fibers From By-Products of Edible Fungi Industry: Basic Structure, Physicochemical Properties, and Their Effects on Energy Intake
Source: Front Nutr. 2022 Mar 10;9:851228. doi: 10.3389/fnut.2022.851228 (PMC8961438; doi:10.3389/fnut.2022.851228)

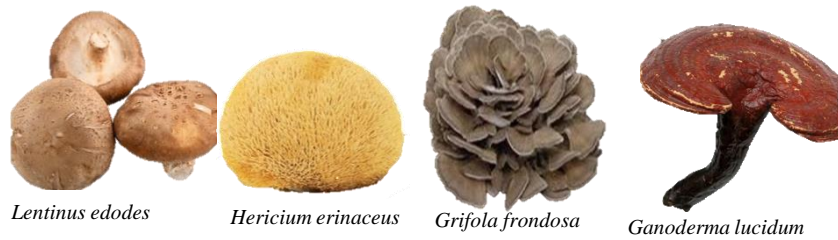

Preparation : AOAC 985.29

Yield

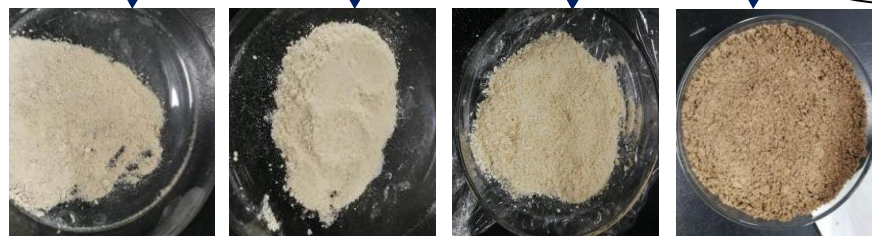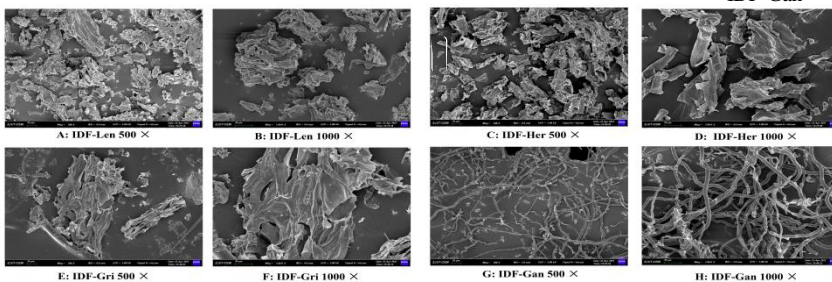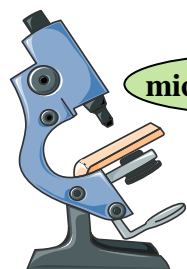

microstructure

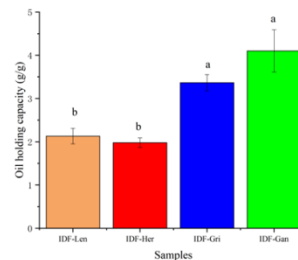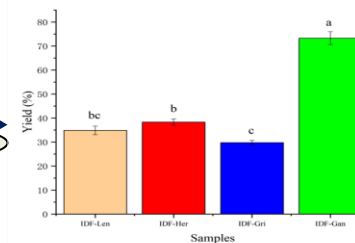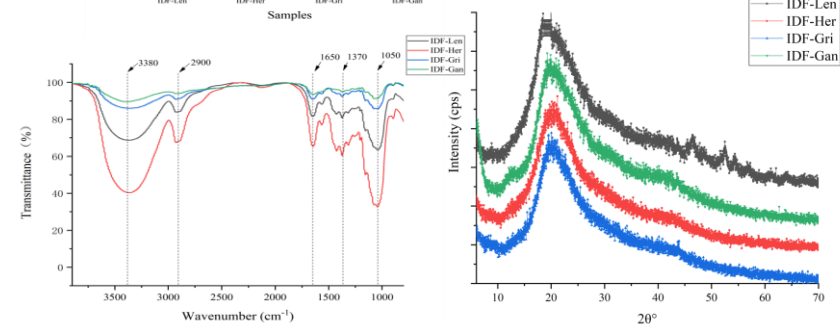

basically structure

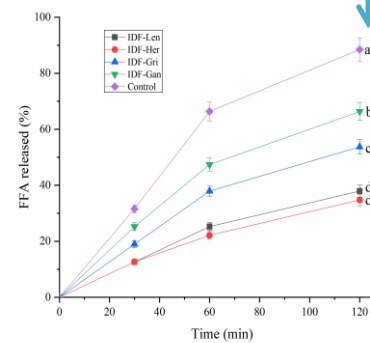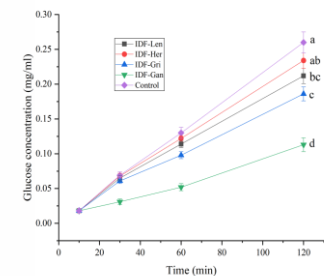

effects on energy intake

Supplement: Supplementary file 1 [file Data_Sheet_1.PDF]
